# Classic Coagulation Traits Vary According to Rh(D) (But Not ABO) Blood Groups

**DOI:** 10.3390/hematolrep17060062

**Published:** 2025-11-15

**Authors:** Gilberto Santos Morais-Junior, Patrícia Dias da Silva, Mayara Barbosa da Silva, Jamila Reis de Oliveira, Andersen Charles Daros, Ciro Martins Gomes, Otávio Toledo Nóbrega

**Affiliations:** Graduate Program in Medical Sciences, University of Brasília, Campus Universitário Darcy Ribeiro, Asa Norte, Brasília 70910-900, DF, Brazil

**Keywords:** coagulation, hemostasis, erythrocyte antigen, blood type

## Abstract

**Background:** This study evaluated possible variations in classic blood coagulation parameters according to groups formed from the main erythrocyte antigen systems. **Methods:** Consecutive patients admitted to a transfusion hemotherapy service at a private hospital in the Brazilian Federal District were evaluated for coagulation profile and blood type according to routine laboratory practices. The international normalized ratio (INR), the activated partial thromboplastin time (APTT) and the prothrombin time (PT) were compared according to the ABO blood group and the Rh factor in analyses controlled for classic influencers such as age, sex and comorbidities. **Results:** No significant differences in coagulation were found between groups defined by the ABO antigen system, despite a body of evidence in favor of this correlation. Rh-positive individuals showed increased mean values in PT (13.7 vs. 12.6 s), in APTT (32.0 vs. 30.1 s) and in INR (1.23 vs. 1.15 s) when compared to the Rh-negative counterparts. **Conclusions:** Our results suggest a lowered rate of coagulation among Rh-positive individuals, possibly owing to inhibitory effects of the Rh(D) erythrocyte antigen on the coagulation pathway.

## 1. Introduction

When discovered by the Austrian scientist Karl Landsteiner in 1900, blood groups A, B and O were defined based on the presence or absence of antigens on the surface of red blood cells, and it was later demonstrated by DesCasterllo and Sturli (1902) [1] that the simultaneous presence of both antigens defined AB blood type. Furthermore, it was then realized that anti-A and anti-B antibodies are developed in the first year of life, normally being the IgM type and induced against food and environmental antigens (including microorganisms), which are very similar in structural properties to the A and B antigens [2,3,4].

Even after the discovery of the ABO group and erythrocyte antigens, Landsteiner and Wiener (1940) [5] observed unusual immunological reactions in patients who had received blood transfusions. Thus, rabbit serum was added to red blood cells from *rhesus* monkeys among attempts to identify alternative erythrocyte antigens, thereby obtaining a reagent capable of agglutinating with red blood cells from 85% of Caucasians independently from the ABO group [5]. These individuals were called Rh positive, in reference to the use of *Rhesus* red blood [6,7], and called Rh factor, Rh agglutinogen or factor D [7].

In addition to their great clinical importance with regard to transfusion success among patients, the ABO and Rh systems have also been studied as interfering factors in other health conditions, such as blood clotting [8,9]. Hemostasis associated with blood coagulation includes the aggregation process of platelets which, when activated, adhere to each other and to the adhesion site. It also includes fibrin deposition, a fibrous protein that polymerizes on platelets, forming a mesh that strengthens and stabilizes the blood clot. Several factors are necessary for the perfect execution of these processes, including Factor VIII and von Willebrand Factor, which play important roles in the entire formation and organization chain of a clot [10,11]. It is well known that the plasma levels of these elements vary depending on age, sex and pregnancy status, as well as other factors [10,11]. Thus, the purpose of this study was to investigate a possible influence of common blood groups (ABO and Rh) on the coagulation profile of adult patients.

## 2. Methods

A cross-sectional observational study was conducted for associative analysis between quantitative data representing the coagulation profile and blood typing of adult patients consecutively admitted to the hemotherapy service of a private hospital in the city of Brasília, Federal District, Brazil. Socio-demographic data such as sex, age, weight and height were obtained from the electronic medical record of the transfusion service. Participants of both sexes aged 18 years or over who attended the health service due to varying clinical criteria, and who became, for any reason, eligible to participate in a transfusion process, whether as a donor or recipient, were included. To assure protection for all volunteers, the trial followed recommendations of the Declaration of Helsinki. All procedures were submitted to and approved by the Research Ethics Committee from the University of Brasilia before implementation (CAAE 70212123.9.0000.8118), approved on date 8 January 2023, with each participant signing an Informed Consent Form. 

Each participant was necessarily subjected to classic coagulation tests and a complete blood count as part of the service’s routine. Individuals under 18 years of age, individuals with any congenital or acquired coagulation disorder (i.e., Von Willebrand’s disease) or patients regularly using interfering drugs (i.e., anticoagulants) were excluded. Comorbidities and pre-existing diseases were identified during a clinical interview based on the patient’s report. Blood samples from recipient patients were collected one month or more after a blood transfusion or when the case was considered resolved according to clinical criteria by the medical team.

Blood typing was performed using a routine technique, with whole blood collected without the need for fasting in a tube with anticoagulant (EDTA) to separate the plasma. Direct typing and reverse typing methods were used based on the principle of red blood cell agglutination due to incompatibility with standard reagent [12,13,14], using BioRad^®^ brand reagents (Hercules, CA, USA). Coagulation tests were performed with a blood sample collected in the presence of sodium citrate to promote clotting and determine the prothrombin time (PT), in seconds [15]. The time required for recalcified plasma to clot in the presence of phospholipid or partial thromboplastin was evaluated using the same sample, a test known as activated partial thromboplastin time (APTT), also measured in seconds [15]. The INR was determined by the ratio of the patient’s PT to the mean normal prothrombin time, raised to an international sensitivity index (ISI) [16] as provided by the thromboplastin manufacturer. LabTest kits were used for coagulation tests (Lagoa Santa, Minas Gerais, Brazil).

Tests to determine serum glycemia, complete blood count, C-reactive protein (C-RP) and thyroid-stimulating hormone (TSH) were performed automatically using a HemaBio III hematological analyzer (Biotécnica, Varginha, Minas Gerais, Brazil), as well as a biochemical analyzer LabMax240 (LabTest, Lagoa Santa, Minas Gerais, Brazil) for the other biochemical tests included in this study.

Statistical analyses consisted of inferential tests of covariance (ANCOVA) or partial correlation test (Pearson’s or Spearman’s), controlled for other dependent variables (covariates) for which correlation tests or inferential tests indicated *p* < 0.06. Data were analyzed using the Statistical Package for the Social Sciences (SPSS) software version 23.

## 3. Results

The present study (Table 1) sought to analyze variables which may influence the general coagulation profile of patients. Our sample consisted of convenience samples with a total of 404 participants, the majority of whom were women (58%). Regarding health conditions, the sample had a mean glycemic value (114.7 mg/dL) compatible with a pre-diabetic condition (100 to 125 mg/dL), as well as a lower mean hemoglobin level (9.2 g/dL) compared to reference values for both men (14 to 18 g/dL) and women (12 to 16 g/dL).

The Student’s *t* test for independent samples was used. Data are expressed as mean ± standard deviation followed by the significance level (*p*-value). ADD = antidiabetic drug; AMD = antimicrobial drug; AP = antiplatelet; APTT = activated partial thromboplastin time; BMI = body mass index; CKD = chronic kidney disease; CNS = central nervous system; COVID-19 = coronavirus disease 2019; C-RP = C-reactive protein; CSD = corticosteroid drug; CVD = cardiovascular drug; ESLD = end-stage liver disease; IBD = immunobiological drug; INR = international normalized ratio; NSAIDs = nonsteroidal anti-inflammatory drug; PT = prothrombin time; SAH = systemic arterial hypertension; T2DM = type 2 diabetes mellitus.

Systemic arterial hypertension stood out among the comorbidities evaluated as the most prevalent condition, followed by type 2 diabetes mellitus, which is in line with the predominance of these chronic conditions in the Brazilian population [17]. A significant consumption prevalence of antihypertensive medications (39.1%) and central nervous system agents (28.9%) was observed, followed by analgesics (22.7%) among the investigated sample.

In general, no significant differences were found in coagulation parameters according to blood groups formed from the ABO system. Table 1 compares clinical characteristics of participants belonging to blood group O with those belonging to the other blood groups combined (A, B and AB) to represent this finding. Even analyses involving other forms of grouping according to the ABO system failed to reveal differences in coagulation parameters.

In contrast, it was observed that the group carrying the Rh antigen showed a slight slowdown in blood clotting, as evidenced by longer mean values in prothrombin time (PT), activated partial thromboplastin time (APTT) and international normalized ratio (INR) among Rh-positive individuals. Average elevations of more than one second in prothrombin time and almost two seconds in prothrombin partial activation time were observed. Platelet levels did not differ between individuals in group O when compared to other groups (isolated or combined) of the ABO system, or according to patients categorized by the Rh system.

Certain comorbidities or previous conditions were observed at different frequencies according to blood groups, such as a significant increase in the prevalence of chronic kidney disease among Rh(D)-positive patients. When compared to all other groups in the ABO system, patients with blood group O showed both an increased prevalence of sepsis and a reduced consumption of antidiabetic medications.

**Figure 1:**
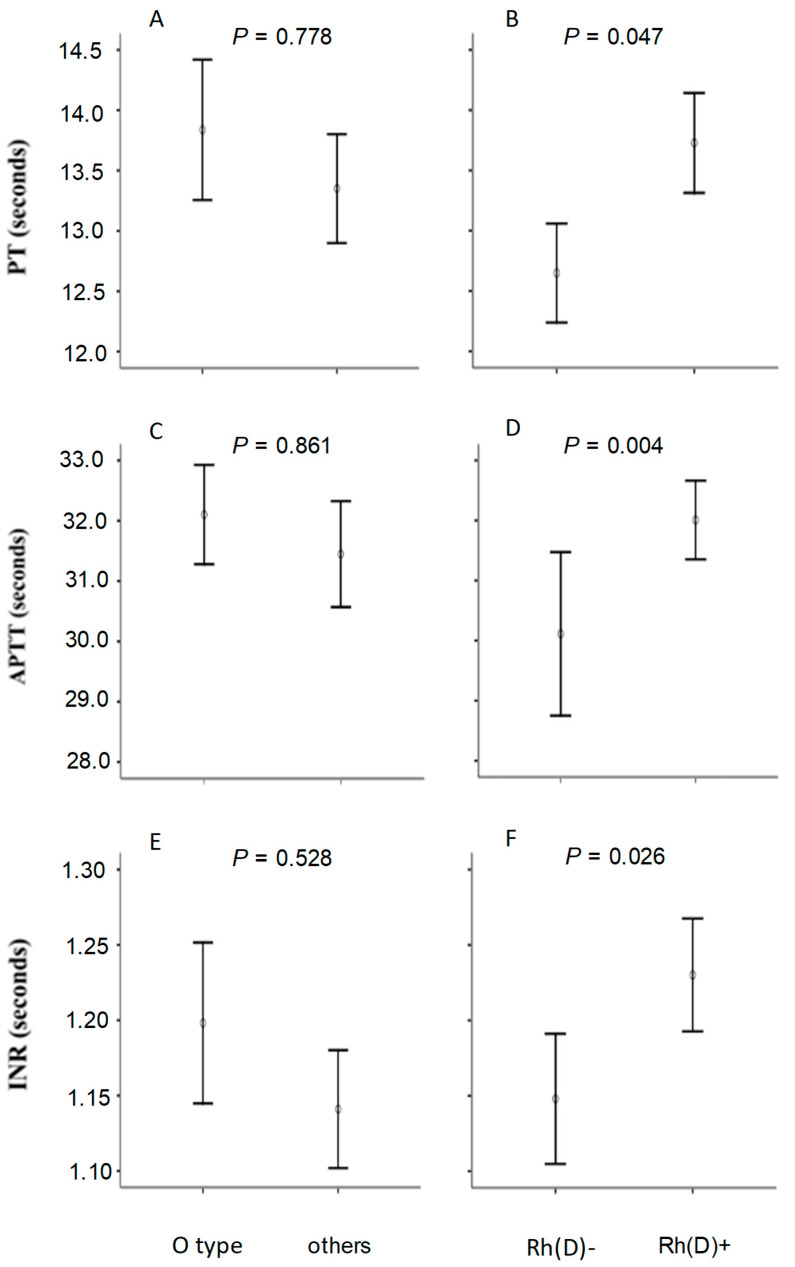
Comparison of mean values of prothrombin time (PT), of activated partial thromboplastin time (APTT) and of the international normalized ratio (INR) according to the ABO and Rh(D) blood group types. Significance was verified by covariance analyses controlled for presence of end-stage liver disease, of sepsis or of antidiabetic drug use (panels **A**, **C** and **E**) and for presence of chronic kidney disease (panels **B**, **D** and **F**). Vertical bars represent intervals of two standard errors.

A complementary analysis compared mean PT, APTT and INR values according to ABO and Rh(D) blood groups, with significance assessed by covariance analysis to control for a possible influence of the unequal frequency of the aforementioned phenotypes. It was possible to notice that both the difference observed in PT, APTT and INR according to Rh(D) groups (Figure 1; panels B, D and F) and the absence of difference between ABO groups (panels A, C and E) remained after the adjustments were made.

## 4. Discussion

Understanding the relationship between erythrocyte antigens and blood clotting is of fundamental importance for hematology and transfusion medicine. Contrarily to the majority of literature [3,18,19,20,21,22,23], no relevant changes were identified in classic coagulometric indices when comparing groups according to the ABO system. On the other hand, comparing the patients according to Rh(D) agglutinogen revealed parameters compatible with delayed coagulation among Rh(D)-positive patients, suggesting possible modulation by this antigenic system. From a clinical point of view, the discrepancies shown in this study in PT, APTT and INR do not reflect a substantial increase that could produce obvious effects on individual outcomes. However, these interpersonal variations may represent an aggravation in the context of primary or secondary coagulation disorders, which may justify additional investigations to elucidate possible underlying mechanisms that support the hypothesis that Rh(D)-positive individuals present a mild delayed coagulation cascade compared to non-carriers of the erythrocyte D antigen.

A study with the Nigerian population [24] appears to corroborate our finding by revealing reduced plasma levels of von Willebrand factor (VWF) among O Rh(D)-positive individuals when compared to Rh(D)-negative ones, to the point of producing a slight slowdown in coagulation tests in patients carrying the D antigen. A study with a population from the Indian subcontinent showed similar results, observing that Rh(D)-positive blood donors exhibited lower VWF levels than Rh(D)-negative donors [18]. The hypothesis that VWF levels vary depending on the presence of the Rh(D) erythrocyte antigen needs to be further explored in the literature, as it can, at least partially, explain the significant variation in coagulatory variables. On the other hand, work carried out in Mediterranean Africa sought to study the coagulation profile according to the ABO and Rh(D) groups among patients with HELLP Syndrome, a specific and rare pregnancy disorder, and found no differences between groups in controlled analyses for interferers [25], suggesting that our finding is not generalizable to different human population groups or that it is sensitive to the context of each group.

Another avenue of explanation for the observed phenomenon consists of the evidence that the Rh(D) erythrocyte antigen non-covalently binds to other elements of the coagulation cascade. One example is CD47, a protein that is not only abundant, but also functionally relevant to platelet homeostasis [26]. It is known that the coagulation cascade is a complex process that involves platelets and other soluble components in the blood, and whose mechanism can be modulated by deficiency in specific factors, platelet disorders and liver diseases, among other factors. The Rh(D) antigen is not known to directly influence these processes, but an indirect influence cannot be ruled out, such as a lower efficiency of platelet activity due to competitive blockade of the CD47 protein by the Rh(D) erythrocyte antigen, or even another antigen with which it presents allelic linkage disequilibrium [27].

Another element of the coagulatory cascade that could be altered is the characteristic glycoprotein of the LW blood group (ICAM-4), which also forms a non-covalent bond with the D antigen. Under physiological conditions, ICAM-4 promotes intercellular adhesion both between platelets and from platelets to erythrocytes, in addition to being a crucial ligand for the platelet fibrinogen receptor (glycoprotein IIb/IIIa), thus contributing to form the hemostatic plug. Therefore, ICAM-4 deficiency due to natural blockade by the D antigen (or another antigen secreted with D) could also result in a reduction in platelet adhesiveness, compromising efficient clot formation [28].

Although this study has advanced understanding of the relationship between ABO and Rh erythrocyte antigens and blood clotting, there are significant limitations. First, our analysis did not involve measuring specific coagulation factors, such as von Willebrand Factor, Factor VIII or other elements of human coagulation. Furthermore, conducting the study with a sample from a single country prevents generalizing the findings to other population groups. The study was conducted on hospitalized patients where there was no single motivation for hospital admission. Furthermore, the fact that they can be blood donors or transfusion recipients adds complexity to the scenario by bringing together participants with different basic clinical conditions, even with the methodological care of only admitting recipients when clinically recovered from the cause that motivated the transfusion event. In line with this, biological interferences and/or lifestyle not considered in the analyses can also be considered as limiting the study, despite the statistical adjustments made.

In this study, we acknowledge that the distribution of participants across ABO and Rh blood groups was not fully balanced, and we did not stratify analyses based on clinical roles such as donors versus recipients. This may have introduced potential confounding effects related to underlying clinical differences between groups. While our primary objective was to explore overall associations between blood group systems and coagulation parameters, we recognize that variations in clinical status, treatment exposure, and comorbidities among participants could have influenced the findings. Therefore, we have identified this as a limitation of the study. Future research with larger, more stratified samples and clinically matched subgroups is needed to better isolate the influence of blood group characteristics from other confounding variables and to validate these associations with greater precision.

Based on the characteristics of the study and the limitations identified, a more comprehensive and rigorous approach is recommended, especially in investigating the relationship between the Rh(D) factor and human blood coagulation processes given the scarcity of evidence in current scientific literature. It is essential to expand the sample to include a representative diversity of participants, detailing the comorbidities of hospitalized patients to consider their possible impact on the study results, as well as interferences caused by specific treatments. In methodological terms, including a more comprehensive analysis of coagulation factors is suggested. Furthermore, longitudinal studies are recommended to investigate changes in coagulation parameters over time and their relationship with comorbidity progression and other clinical factors. These recommendations have the potential to guide more robust and informative future research into the relationship between blood groups and blood clotting.

## 5. Conclusions

An association between blood group typing and coagulation profiles was identified, revealing that the Rh factor may exert a deleterious influence on coagulation parameters, specifically, Rh-positive individuals demonstrated significantly prolonged prothrombin time (PT), activated partial thromboplastin time (APTT) and international normalized ratio (INR) values compared to Rh-negative individuals. This study examined the influence of multiple variables, including ABO blood group, Rh factor, sex, body mass index (BMI), age and comorbidities such as diabetes mellitus and systemic arterial hypertension, on blood coagulation dynamics. The observed correlation between the Rh factor and altered coagulation test results underscores the importance of considering not only the ABO blood group, but also Rh status in the assessment of hemostatic function. These findings contribute to the growing recognition of the impact of human biological diversity on physiological processes and support the need for a more individualized approach to clinical evaluation. Nevertheless, we acknowledge that further rigorous, multidisciplinary investigations are essential to fully elucidate these complex interactions and their potential clinical implications, ultimately aiming to enhance patient care and population health outcomes.

## Figures and Tables

**Table 1:** Comparison of clinical, biochemical and coagulation traits according to the ABO and Rh(D) blood group types among the 404 adults investigated.

	*Blood Group Systems*					
	ABO				Rh (D)	
	O Type (n = 209)	Others (n = 195)	*p*-Value	Negative (n = 47)	Positive (n = 357)	*p*-Value
Age (years)	54.8 ± 20.8	57.9 ± 20.5	0.140	52.6 ± 19.9	56.8 ± 20.8	0.190
Sex (% male)	41.1	42.6	0.840	46.8	41.2	0.530
BMI (kg/m^2^)	25.5 ± 4.8	25.6 ± 4.7	0.736	26.3 ± 5.2	25.4 ± 4.7	0.300
Hemoglobin (g/dL)	9.1 ± 1.4	9.3 ± 1.4	0.289	9.6 ± 1.6	9.1 ± 1.4	0.086
Leucocytes (10^3^/mm^3^)	10.0 ± 6.8	9.5 ± 6.8	0.422	9.6 ± 6.0	9.7 ± 6.9	0.845
Glucose (mg/dL)	113.4 ± 39.4	116.9 ± 45.2	0.401	125.5 ± 53.7	113.7 ± 40.4	0.151
C-RP (mg/dL)	5.0 ± 2.2	5.0 ± 2.3	0.815	5.4 ± 2.6	4.9 ± 2.1	0.566
PT (sec)	13.8 ± 4.2	13.4 ± 3.2	0.191	12.6 ± 1.4	13.7 ± 3.9	< 0.001
APTT (sec)	32.1 ± 6.0	31.4 ± 6.1	0.278	30.1 ± 4.7	32.0 ± 6.2	0.043
INR (index	1.25 ± 0.39	1.19 ± 0.27	0.089	1.15 ± 0.15	1.23 ± 0.35	0.005
N/P ratio	1.12 ± 0.23	1.09 ± 0.22	0.189	1.06 ± 0.17	1.12 ± 0.23	0.087
Platelet (10^3^/mm^3^)	2.34 ± 1.11	2.12 ± 1.04	0.212	2.51 ± 1.36	2.20 ± 1.04	0.145
SAH (% with)	37.7	42.6	0.360	48.9	38.7	0.364
CKD (% with)	15.3	11.3	0.234	4.3	14.6	0.051
ESLD (% with)	2.9	0.0	0.017	0.0	1.7	0.371
COPD (% with)	7.7	7.2	0.855	6.4	7.6	0.772
T2DM (% with)	28.2	34.4	0.198	23.4	32.2	0.245
SEPSIS (% with)	17.2	9.7	0.028	8.5	14.3	0.278
DENGUE (% with)	1.4	1.0	0.710	0.0	1.4	0.414
COVID-19 (% with)	2.9	6.2	0.110	2.1	4.8	0.411
NSAID use (% yes)	21.1	24.1	0.463	19,1	23.0	0.556
CSD use (% yes)	8.6	7.2	0.594	6.4	8.1	0.678
AMD use (% yes)	24.4	21.0	0.419	23.4	22.7	0.912
IBD use (% yes)	4.3	9.2	0.068	6.4	6.7	0.930
ADD use (% yes)	16.7	28.7	0.004	17.0	23.2	0.337
CVD use (% yes)	35.9	42.6	0.169	31.9	40.1	0.282
CNS agent use (% yes)	24.9	33.3	0.061	38.3	27.7	0.133
AP agent use (% yes)	3.3	7.2	0.083	4.3	5.3	0.757

## Data Availability

All data generated with this study is included in the publication, with raw analyses and materials being available upon a reasonable request.
